# The F‐Actin of In Situ Chondrocytes in Non‐Degenerate and Mildly‐Degenerate Human Cartilage: Changes With Cell Volume, and Cytoplasmic Processes

**DOI:** 10.1002/jor.70303

**Published:** 2026-10-22

**Authors:** Mohammed S. M. Al Garni, Anish K. Amin, Andrew C. Hall

**Affiliations:** ^1^ Biomedical Sciences, Centre for Discovery Brain Sciences University of Edinburgh Edinburgh UK; ^2^ Department of Basic Medical Sciences College of Medicine University of Jeddah Jeddah Saudi Arabia; ^3^ Edinburgh Orthopaedics, Royal Infirmary of Edinburgh Edinburgh UK

**Keywords:** chondrocyte, human articular cartilage, morphology, osteoarthritis, phenotype

## Abstract

The F‐actin cytoskeleton regulates chondrocyte morphology, cell–matrix interactions, and phenotype. Abnormal chondrocyte properties observed in non‐degenerate human cartilage suggest early phenotypic de‐differentiation that may contribute to weakening of the extracellular matrix. This study investigated the relationships between in situ chondrocyte volume, clustering, morphology, and F‐actin organization, and examined whether the formation of cytoplasmic processes was an active cellular event. Fresh ex vivo human femoral head cartilage explants from 22 patients were graded individually as non‐degenerate (G0) or mildly degenerate (G1). Explants were labeled with Cell Tracker and phalloidin to visualize chondrocytes and F‐actin, respectively, and imaged using confocal laser scanning microscopy. G1 explants were additionally exposed to hypo‐osmotic pressure (170 mOsm, 10 min) to assess changes to the morphology of the cytoplasmic processes. Large chondrocytes with spheroidal morphology demonstrated increased F‐actin levels and redistribution in both G0 and G1 cartilage (*p* < 0.05). Clustered chondrocytes exhibited significantly increased F‐actin content and altered distribution with degeneration (*p* ≤ 0.0004). Chondrocytes possessing cytoplasmic processes exhibited marked focal F‐actin accumulation within the extensions (*p* < 0.0001). In G0 cartilage, F‐actin localized predominantly at the base of processes, whereas in G1 cartilage, it extended throughout both the base and body of the process. Hypo‐osmotic challenge significantly increased chondrocyte volume and process width (*p* < 0.01; *p* < 0.0001), but not process length, despite clear swelling of the cell body. These findings demonstrated that active cytoskeletal F‐actin remodeling may accompany early chondrocyte morphological changes associated with human cartilage degeneration, as occurs in osteoarthritis.

## 1 Introduction

Chondrocytes are the principal cell type in articular cartilage and are exclusively responsible for the maintenance of the resilient extracellular matrix (ECM) by their control of matrix turnover [1, 2] over many decades. They are sparsely distributed (5%–10% of tissue volume), and the stable phenotype is crucial for optimal metabolism of the wide range of complex matrix macromolecules [3] so as to prevent focal areas of weakness potentially leading to cartilage degeneration and osteoarthritis (OA) [4]. The maintenance of the chondrocytic phenotype is also essential in connective tissue engineering approaches [5] so that a resilient hyaline ECM is produced. There are many examples where the loss of the chondrocyte phenotype leads to the production of mechanically incompetent fibro‐cartilaginous matrix constituents, cell senescence, programmed and non‐programmed cell death and changes to signal transduction pathways associated with OA and cartilage ageing [6, 7, 8]. Changes to chondrocyte phenotype may be due to pro‐inflammatory cytokines interleukin‐1β (IL‐1β) and tumor necrosis factor–α (TNF‐α) which have been implicated as mediators of OA pathogenesis [9]. This has been shown to induce chondrocyte de‐differentiation, morphological changes and proliferation because of alterations to the F‐actin cytoskeleton and associated signaling pathways leading to the production of classical fibro‐cartilagenous constituents (e.g., Type I collagen) [10, 11, 12, 13]. An understanding of the F‐actin content and its distribution within in situ human chondrocytes [14] may give valuable fundamental information about the control of normal matrix metabolism in healthy cartilage and its breakdown in OA.

From high‐resolution confocal images obtained from fluorescently‐labeled in situ chondrocytes, the surface of “normal” (rounded/elliptical) chondrocytes appears to be relatively “smooth.” However, it is now established that in human femoral head and tibial plateau cartilage, there is a significant proportion of chondrocytes with fine cytoplasmic processes of 5–15 µm which extend from the cell body into the bulk of the ECM [14, 15]. By studying fluorescently‐labeled in situ human chondrocytes using confocal laser scanning microscopy (CLSM) and imaging, these chondrocyte extrusions have been observed in macroscopically normal (grade 0 (G0) – non‐degenerate) and mildly‐degenerate (grade 1) femoral head and tibial plateau cartilage [14, 15]. In G0 cartilage, a small (10%–15%) but potentially important population of chondrocytes has been reported with abnormal morphology. These cytoplasmic processes are completely different from the short (1–2 µm) single cilium present in each chondrocyte, which has been identified in human and animal hyaline cartilage chondrocytes and which requires specific labeling of the tubulin structure for their identification [16, 17]. Other changes to chondrocytes have been identified with cartilage degeneration, in particular the significant increase in cell volume with cartilage degeneration [15, 18] as well as the phenomenon of chondrocyte proliferation (“clustering”) which is particularly evident near tissue fissures [14, 19]. These alterations to normal in situ chondrocyte shape, volume, and proliferation in non‐degenerate human articular cartilage are of special interest because of the strong relationship between the loss of the chondrocyte phenotype leading to localized deleterious changes in matrix metabolism and thus cartilage degeneration [10, 20].

Any changes to chondrocyte properties (volume, morphology, clustering) will involve marked changes to the cytoskeleton – in particular, F‐actin, which is a major determinant of the morphology and volume in many cell types, including chondrocytes [12, 21, 22]. Careful previous studies demonstrate that chondrocytes within the superficial, transitional, and deep zones of healthy articular cartilage exhibit a predominantly cortical F‐actin organization, with higher cytoskeletal staining intensity in the superficial zone (SZ), probably reflecting differences in the local mechanical environment rather than major differences in actin architecture [21]. In isolated chondrocytes, rapid (seconds to minutes) hypo‐osmotic challenge leads to increased cell volume, decreased F‐actin levels and a more diffuse F‐actin distribution [23, 24, 25]. The cellular response to hypo‐osmotic challenge involves transmembrane channels (e.g. TRPV4 and AQP1), Ca²⁺ mobilisation and subsequent cell volume recovery [26, 27]. However, in isolated and in situ chondrocytes, IL‐1β inhibits the transient increase in Ca²⁺ and suppresses cell volume recovery following hypo‐osmotic challenge. The results suggested that remodeling of the F‐actin cytoskeleton was mediated by Rho family GTPases [28, 29]. Other changes to the F‐actin cytoskeleton occur during chondrocyte “clustering” (i.e., proliferation). The expression of genes which promote proliferation (e.g., cyclin D1 (CCND1) and Ki‐67 (Ki67)) increases in parallel with F‐actin polymerization during bovine chondrocyte passaging [13]. Additionally, actin depolymerization reduces the gene expression of (CCND1) and (Ki67) in chondrocytes [9, 13] and other cell types [30, 31], supporting the important active role of the F‐actin cytoskeleton in regulating cell proliferation and other chondrocyte properties. However, the role of F‐actin in the formation and development of the cytoplasmic processes in human articular cartilage chondrocytes is currently unknown. This is potentially important because the role of F‐actin in chondrocyte volume, morphology, and thus phenotype may help understand the deleterious changes to chondrocyte matrix metabolism, which might underpin the initiation and early development of OA.

The presence of cytoplasmic processes in in situ human chondrocytes [15, 20] is intriguing, and studying the F‐actin content and its distribution could clarify whether their formation develops either “passively” or “actively.” The “passive” expansion of the cell cytoplasm into the ECM could arise following mechanical injury, and/or the loss of matrix components, perhaps as a result of the aging process [32]. There are many complex changes to the ECM with aging and also potentially inflammatory episodes [33], which might leave a long‐lasting detrimental effect on cartilage properties. For example, aged cartilage loses aggrecan as the biosynthetic capacity of cartilage declines after skeletal maturity, and extensive post‐translational modification of aggrecan occurs [34]. This markedly affects the pattern and sulphation of GAG chains and their protein core, which will profoundly alter the tissues' mechanical resilience and hence the chondrocyte's environment. Thus, it is possible that the development of chondrocyte processes could be an event resulting from chondrocytes “passively” expanding/swelling into the pericellular/ECM [35], which is defined biochemically by several molecular components, including type VI collagen and perlecan [36].

Alternatively, the cytoplasmic processes could be the result of an “active” episode. This could be where, following activation of intracellular signaling mechanisms, cytoskeletal elements, particularly F‐actin, are stimulated to produce and develop the structure of the cytoplasmic process. This may be associated with the release of matrix‐degrading enzymes, leading to local microstructural weakness, permitting the extension of the process into the otherwise impenetrable matrix. Such cellular behavior is similar to the migratory process occurring in a wide range of other cell types following stimulation by pro‐inflammatory cytokines, for example, IL‐1, IL‐6, and TNF‐α [37].

To clarify the relationship between the F‐actin cytoskeleton and chondrocyte volume, clustering, and morphology, we have fluorescently labeled the cytoplasmic space and F‐actin of in situ human chondrocytes within the SZ of non‐degenerate and mildly degenerate human femoral head cartilage. The cells were then visualized using CLSM, with the images analyzed for F‐actin content and distribution. The relationship between F‐actin and (a) chondrocyte swelling, (b) cell clustering, and (c) cells exhibiting cytoplasmic processes has been investigated. Furthermore, to determine if the cytoplasmic processes arise “simply” from “passive” cell swelling, we have subjected the in situ cells to a hypo‐osmotic challenge. The results demonstrated marked changes to F‐actin associated with chondrocyte volume, clustering, and morphology, with evidence suggesting that the formation of the cytoplasmic processes is an “active” rather than a “passive” event.

## 2 Materials and Methods

### 2.1 Human Femoral Heads

Ethical approval for 22 femoral heads was obtained (Ref. no.: SR1295 AM02) (Tissue Governance, National Health Service, Lothian) with the patient's consent. The cohort comprised 14 females, 8 males, with a mean age of 69 years (range: 40–90 years), undergoing surgery for femoral neck fracture. No additional patient demographic or clinical information was available as the samples were anonymized. The heads were placed in Dulbecco's Modified Eagle's Medium (DMEM; 340 mOsm) containing 100 U/mL penicillin, 100 μg/mL streptomycin, 25 mg/mL amphotericin B (Sigma‐Aldrich, Irvine, UK), and 10 μg/mL Fungin (InvivoGen, Toulouse, France) and delivered to the laboratory within 2 h.

### 2.2 Cartilage Grading, Harvesting, Fluorescent Labeling of In Situ Chondrocytes, and CLSM

Grading was performed following Osteoarthritis Research Society International criteria [38], and individual explants categorized as described as grade 0 (G0, non‐degenerate) or grade 1 (G1, mildly‐degenerate). Explants were harvested from the circumferential region of the femoral head, distant from the ligamentum teres, with three explants obtained from each grade (G0 and G1) per femoral head. Full depth cartilage explants (without sub‐chondral bone) were incubated with 5‐chloromethylfluorescein diacetate (CMFDA; Cell Tracker green) (12.5 μM; Invitrogen, Paisley, UK) (1.5 h; 21°C) to label the cytoplasmic space of living cells, followed by washing in PBS (Invitrogen) and fixing with 4% formaldehyde (30 min; Fisher). For F‐actin labeling, the explants were permeabilized and stained with fluorescent phalloidin [39] conjugate (Abcam, Cambridge, UK) in PBS‐Tween (1:500 μg/mL; Abcam, Cambridge, UK) (2 h; 21°C), and then washed (PBS‐Tween; 15 min; 3×). In situ SZ chondrocytes within the first ~100 μm were imaged axially using standard settings for CLSM with sequential z‐stack images of 5 and 1 µm, respectively, for low and high magnification as previously described [14].

### 2.3 In Situ Chondrocyte Morphology and Analysis of F‐Actin Content and Distribution

Image analysis was conducted using Imaris (Version 9.3.1; Bitplane) and ImageJ/FIJI (NIH). Chondrocyte characteristics (volume, clustering, processes), together with the fluorescent labeling of the F‐actin cytoskeleton in the same cells, were assessed in G0 and G1 cartilage, within a standardized image size (1024 × 1024 pixels). Chondrocyte volumes were determined utilizing the IMARIS “Surfaces” algorithm, which created an artificial “surface” on individual fluorescently‐labeled chondrocytes. This allowed the “visualisation” of the morphological properties of the cells and was important for assessing the cytoplasmic processes and cell volume. Note that the algorithm does not refer to the depth from the cartilage surface. Calibration of cell volume was performed using fluorescent microspheres (Polysciences, Warrington, PA, USA) following an established protocol [40]. The evaluation of chondrocyte properties involved the visual assessment of (a) “normal” (rounded/elliptical) chondrocytes, (b) chondrocyte clusters (≥ 3 cells/lacuna), and (c) chondrocytes displaying one or more cytoplasmic processes [14] and related these properties to phalloidin‐labeled F‐actin. Chondrocytes were categorized as either in the “normal” volume range (< 1000 µm^3^) or “large” (> 1000 µm^3^) [15]. F‐actin was analyzed as (a) the phalloidin intensity around the cortical ring (Figure 1A – (a–c); Figure 1B), (b) the presence of focal areas of intense punctate labeling (IP; ≤ 2 µm in length), and (c) broader areas of intense labeling (IAs; > 2 µm in length) (Figure 1A – (c); Figure 1B). For clustered chondrocytes, it was not possible to measure the phalloidin labeling level accurately in individual cells as they were frequently touching/overlapping. Therefore, the F‐actin level was measured for the whole cell group and then divided by the number of cells/cluster. In addition, because of the difficulty of accurately identifying the cortical ring of F‐actin, the distribution of F‐actin was expressed as the number of intense “spots”/cell. For chondrocytes with abnormal morphology, the F‐actin within the cell body and cytoplasmic processes was assessed separately. The protrusion of cytoplasmic processes by in situ chondrocytes was categorized into three groups based on their length (L) (L1 < 5 µm, 5 ≥ L2 < 10 µm, L3 ≥ 10 µm), permitting a more detailed quantitative comparison of the F‐actin level and localization. This systematic approach allowed the elucidation of the intricate association between F‐actin distribution and chondrocyte characteristics between the elliptical, “flattened” cells within the SZ of G0 and G1 cartilage. For the F‐actin within the cytoplasmic processes, each process was divided into three regions: base, body, and tip (Figure 1A – (f)). The accumulation of F‐actin in each of these regions was expressed as a percentage (0%, 33%, 50%, and 100%) of the total amount of F‐actin in the *whole* cytoplasmic process. Thus, (i) 0% indicated that there is no accumulation within the cytoplasmic process/es, (ii) 33% showed that the accumulation was observed in the all three regions (base, body, and tip), (iii) 50% indicated that the accumulation appeared in two regions of the cytoplasmic process/es, finally, (iv) 100% indicated that the accumulation was present in one region of the cytoplasmic process/es.

**Figure 1:**
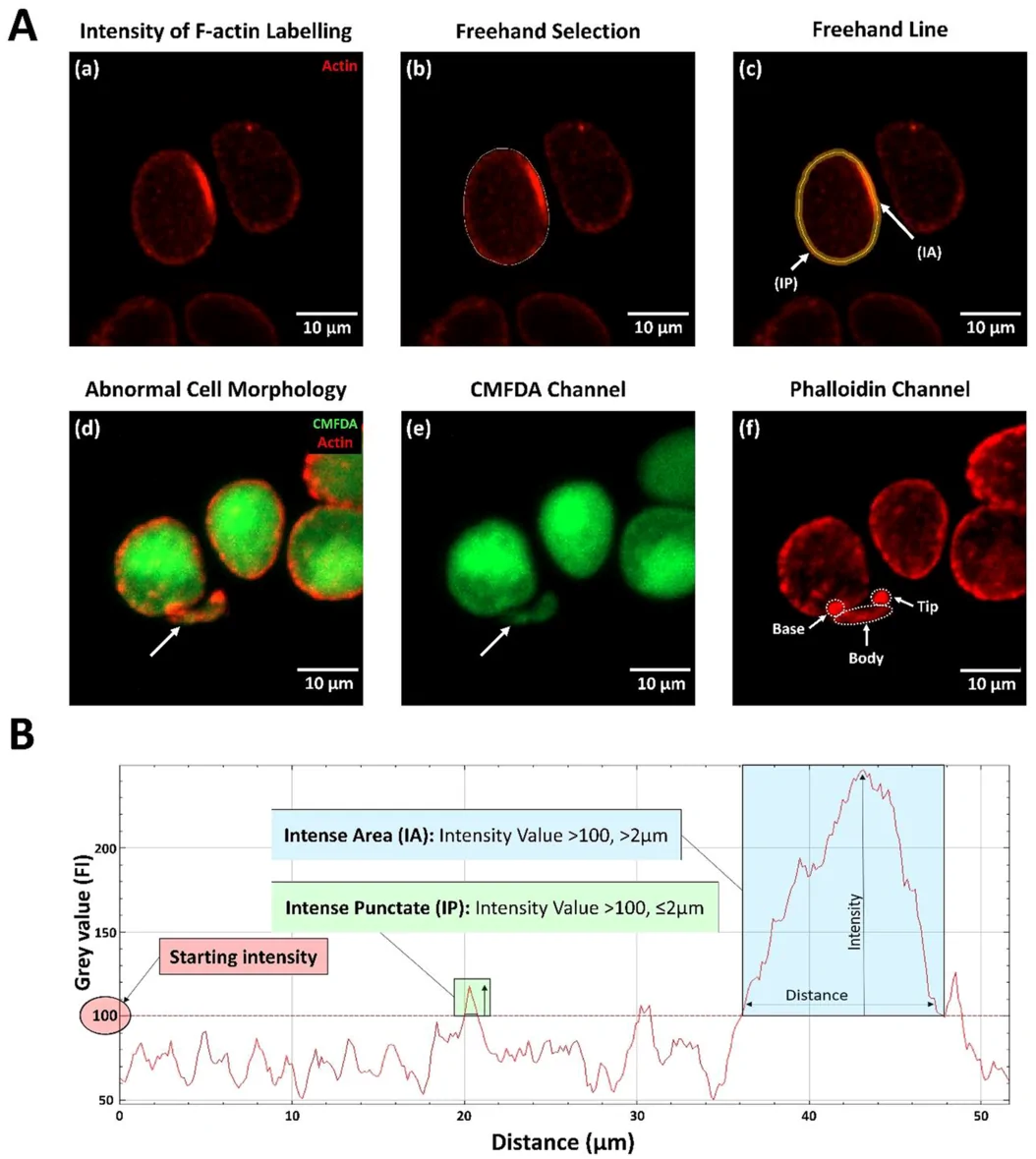
Examples of phalloidin‐labeled F‐actin fluorescence within in situ human chondrocytes. (A) represents high power magnification images (a–c) of in situ chondrocytes illustrating (a) cortical F‐actin, (b) tracing and measurement of average F‐actin fluorescence, using the Freehand selection (thin yellow broken line), and (c) intensity of F‐actin, using Freehand line tools in ImageJ. Examples of the intensity were shown in (c) as intense punctate (IP) and intense areas (IAs). Examples of cytoplasmic process/es (d) and (e), with the classification of F‐actin labeling localization (f), phalloidin‐labeling was assessed on a projected 3D image, highlighting the cortical ring associated with the cell membrane. Bar = 10 µm. Panel (B) shows the fluorescence profile along the cortical region in (a). The red line identified fluorescence levels around the cortical ring. The red circle indicated the background fluorescence level (gray value = 100 fluorescence units (FI)). The green rectangle represented a zone where the fluorescence was above this value for ≤ 2 µm (termed “intense punctate” (IP) area). The blue rectangle represented a region where the fluorescence value was ≥ 100 FI over > 2 µm of the cortical ring (termed “intense area” (IA)).

### 2.4 The Response of Chondrocyte Processes to Hypo‐Osmotic Challenge

To assess whether cytoplasmic protrusions were an “active” or “passive” process, six explants of mildly degenerate (G1) human articular cartilage were obtained from three fresh femoral heads. In situ chondrocytes were labeled with CellTracker Green CMFDA, and an edge of the explant was secured to the Petri dish using Superglue. Chondrocyte volume, and the width and length of cytoplasmic process/es were recorded using CLSM at the control osmotic pressure (DMEM; 340 mOsm; 21°C). To apply the hypo‐osmotic condition, the medium was very carefully removed using a plastic pipette while the dish remained on the microscope stage, ensuring consistent positioning and imaging of the cells under study, and replaced with the hypo‐osmotic medium (170 mOsm; DMEM:distilled water 1:1). Within a few seconds, the same cells were imaged and to distinguish the difference in chondrocyte properties after hypo‐osmotic pressure, the green color of the CMFDA‐stained chondrocytes was changed to red using ImageJ software (Image – Lookup tables – Red). After 10 min incubation, images were acquired from the same region of interest, on the same cells, to assess changes in chondrocyte volume and the dimensions of the cytoplasmic processes.

### 2.5 Data Presentation and Statistical Analysis

Graphs and statistical assessments were conducted using GraphPad Prism ver.9.3.1 (GraphPad, La Jolla, USA). Data were shown as *N*(*n*) with *N* representing the number of independent femoral heads and (*n*) the number of replicates, respectively, from each experiment. For volume measurements, data on individual cells were presented as mean ± SD. This approach was chosen because averaging cell volumes per explant would assign equal weight to explants containing unequal numbers of cells, potentially leading to a misleading representation of the underlying cell population. Explants with different cell numbers and distinct volume distributions would therefore contribute equally, potentially masking biologically‐relevant cell‐to‐cell variability. SD therefore accurately represents variability at the single‐cell level. Cells were analyzed across multiple independent explants to ensure biological replication. Morphology and clustering data were quantified as percentages per explant and are presented as mean ± SEM. Paired or unpaired Student's *t*‐tests were applied for data pairs, whereas ANOVAs with Tukey's *post hoc* correction were used for multiple comparisons. An asterisk (*) indicated a significant difference between groups within the same cartilage grade, whereas a hash sign (#) indicated a significant difference in variables between the cartilage grades. Single, double, triple and quadruple symbols showed the level of significance (*p* < 0.05, 0.01, 0.001, 0.0001, respectively).

## 3 Results

### 3.1 F‐Actin Distribution in Chondrocytes of Normal Morphology and Increased Volume

Axial images of CMFDA and phalloidin‐labeled in situ chondrocytes identified the normal rounded morphology and F‐actin labeling of cells within G0 (Figure 2A – (a,b)) and G1 cartilage (Figure 2A – (d,e)). Higher‐powered images showed that while in the majority of cells, F‐actin was evenly distributed around the cell periphery, in both G0 cartilage and in up to 30% of the cells in G1 cartilage, the F‐actin appeared punctate with discrete areas of increased fluorescence (Figure 2A – (b,e)).

**Figure 2:**
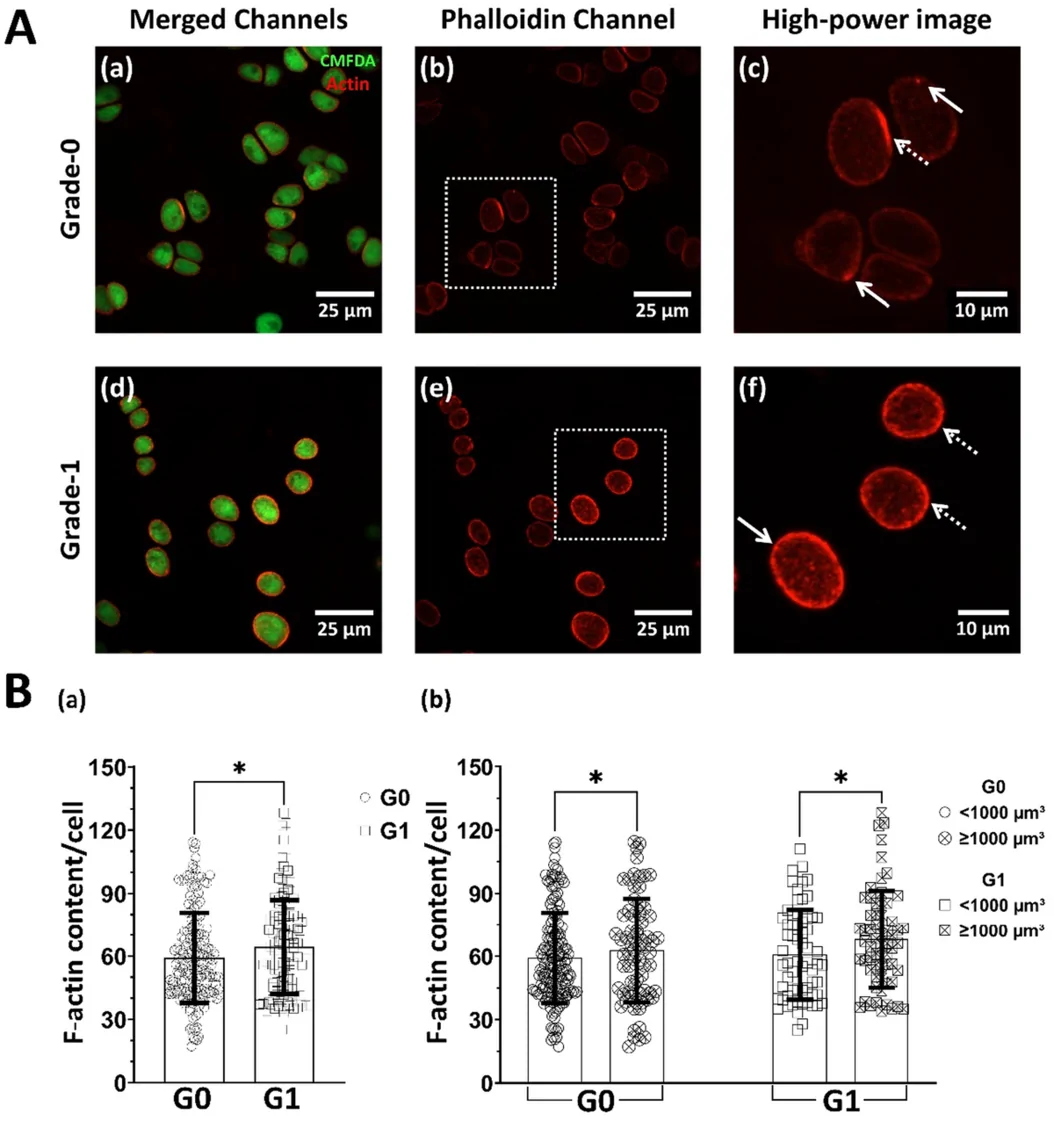
CMFDA and F‐actin in chondrocytes of normal or increased volume within G0 and G1 human cartilage. Panel (A) shows images at low and high magnification of CMFDA (green) and F‐actin (red) labeling of in situ chondrocytes within G0 (a–c) and G1 (d–f) cartilage. Arrows identified areas of F‐actin corresponding to intense areas (IAs; broken line) or intense punctate (IP; solid line; see Figure 1 for details). Panel (B) (a) shows a significant increase in all cells for cortical F‐actin in chondrocytes within G0 and G1 cartilage. Panel (B) (b) shows the significant increase in F‐actin for chondrocytes in the normal volume range (≤ 1000 μm^3^), with those of increased volume (> 1000 μm^3^) for both G0 and G1 cartilage. Data were from G0 *N*(*n*) = 17(165); normal = 12(104); large = 16(61), and G1 *N*(*n*) = 16(124); normal = 11(62); large = 12(62). Data analysis and symbols here and in subsequent figures were as described in Section 2.

Total F‐actin content in the cortical region was increased in cells within G1 cartilage compared to G0 cartilage (59.3 ± 21.5 FI to 64.5 ± 22.4 FI; *p* = 0.0227; Figure 2B – (a)). When the cell populations of G0 and G1 cartilage were separated into those of relatively normal (≤ 1000 μm^3^) or larger volume (> 1000 μm^3^), the F‐actin content was greater in large cells for both G0 and G1 cartilage (*p* = 0.039; *p* = 0.034, respectively; Figure 2B – (b)). These results demonstrated that in situ chondrocytes of increased volume in both G0 and G1 cartilage had significantly higher levels of F‐actin.

F‐actin distribution in single cells was further determined as being within IPs or IAs using the analysis method described in Figure 1. The number of IPs per cell (IPs/cell) increased between G0 and G1 cartilage (2.0 ± 0.2 vs. 2.8 ± 0.3; *p* = 0.008; Figure 3A – (a)). There was also a highly significant increase in the number of IPs/cell in swollen cells in both G0 and G1 cartilage (*p* < 0.0001 for both; Figure 3A – (b)). The number of IAs per cell (IAs/cell) was also significantly higher for cells in mildly‐degenerate (G1) cartilage (1.1 ± 0.1 vs. 1.6 ± 0.1; *p* = 0.0004; Figure 3B – (a)). There were also significant increases in the number of IAs/cell for swollen cells in G0 and G1 cartilage (*p* < 0.0001; *p* < 0.0001, respectively, Figure 3B – (b)). In addition, for swollen cells, the number of IAs/cell increased within both G0 and G1 cartilage compared to cells of normal volume (*p* = 0.0003; *p* = 0.0017, respectively; Figure 3B – (b)). Overall, these results demonstrated that for chondrocytes of normal morphology, there were increases in the total F‐actin level/cell, IPs/cell, and IAs/cell with early cartilage degeneration, and all these properties of F‐actin were greater in large cells in both G0 and G1 cartilage.

**Figure 3:**
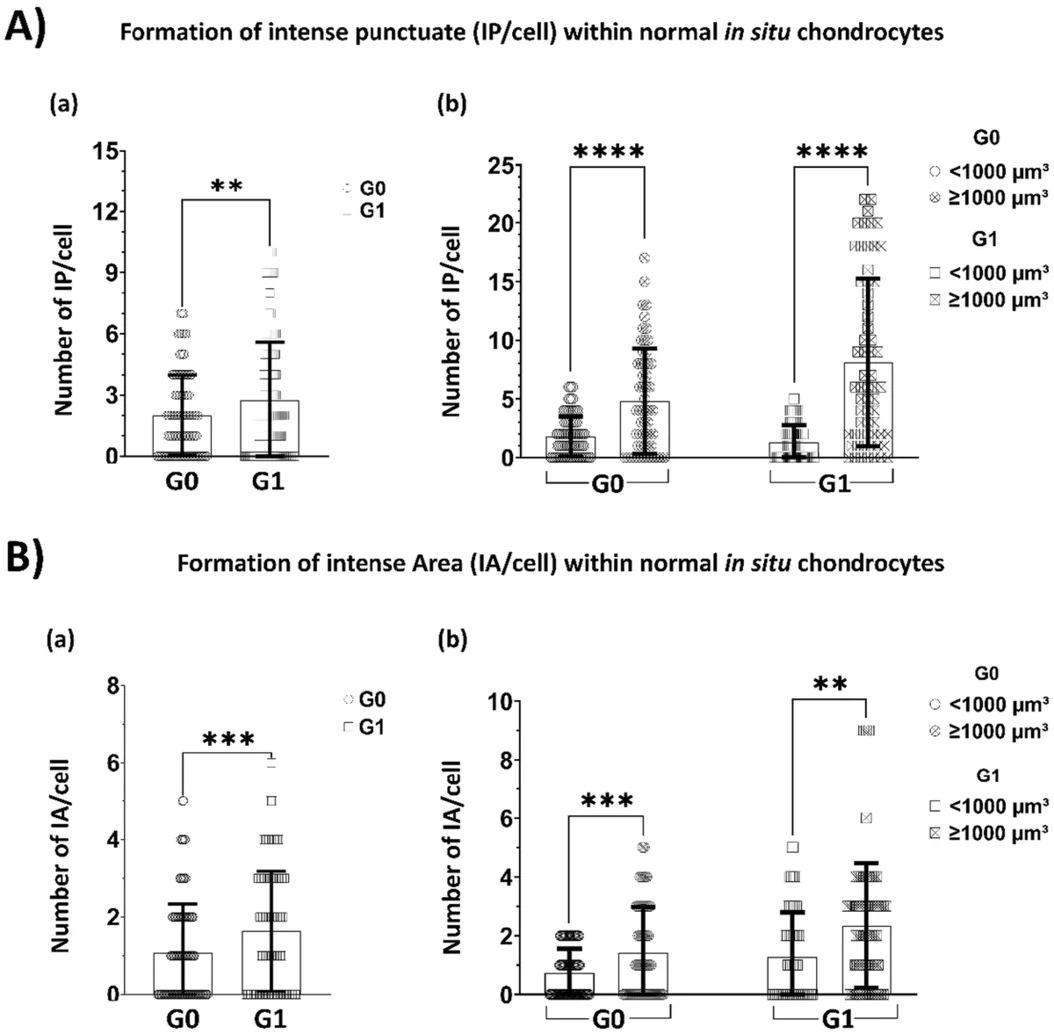
Intense punctates (IP) and intense areas (IA) of F‐actin in normal or large chondrocytes within G0 and G1 human cartilage. Panel (A) (a) shows the significant increase in IP labeling of all chondrocytes between G0 and G1 cartilage. Panel (A) (b) demonstrates the highly significant increase in IP labeling when the chondrocytes were separated into cells with their volume in the lower range (≤ 1000 μm^3^) or with an increased volume (> 1000 μm^3^). Panel (B) (a) shows the significant increase in IA labeling of all chondrocytes studied between cells in G0 and G1 cartilage. Panel (B) (b) demonstrates the highly significant increase in IA labeling between chondrocytes when separated into cells with their volume in the “normal” volume range (≤ 1000 μm^3^) or with an increased volume (> 1000 μm^3^). Data were from G0 – *N*(*n*) = 17(165); normal = 12(104); large = 16(61), and G1 – *N*(*n*) = 16(124); normal = 11(62); large = 12(62).

### 3.2 Chondrocytes in Clusters

Although infrequent, chondrocyte clustering in otherwise non‐degenerate (G0) human femoral head cartilage was easily identified using the cell labeling and confocal approaches described here and in a previous study [14]. In situ chondrocytes, which formed clusters in both G0 and G1 cartilage, displayed marked heterogeneity in F‐actin levels and its distribution (Figure 4A – (a,d)). Axial images of both low (Figure 4A – (a,b,d,e)) and high (Figure 4A – (c,f)] magnification of both grades, showed increased F‐actin content for in situ chondrocytes within G1 compared to chondrocytes in G0 tissue. The overall F‐actin level and the number of intense F‐actin “spots”/cell (see Section 2) within cells forming clusters in G0 and G1 cartilage were increased by > 1.35‐fold (*p* = 0.0004; *p* < 0.0001, respectively). These results suggested that for chondrocytes in clusters, there was a marked increase in the total actin level per cell, and the number of F‐actin “spots” per cell with increasing cartilage degeneration.

**Figure 4:**
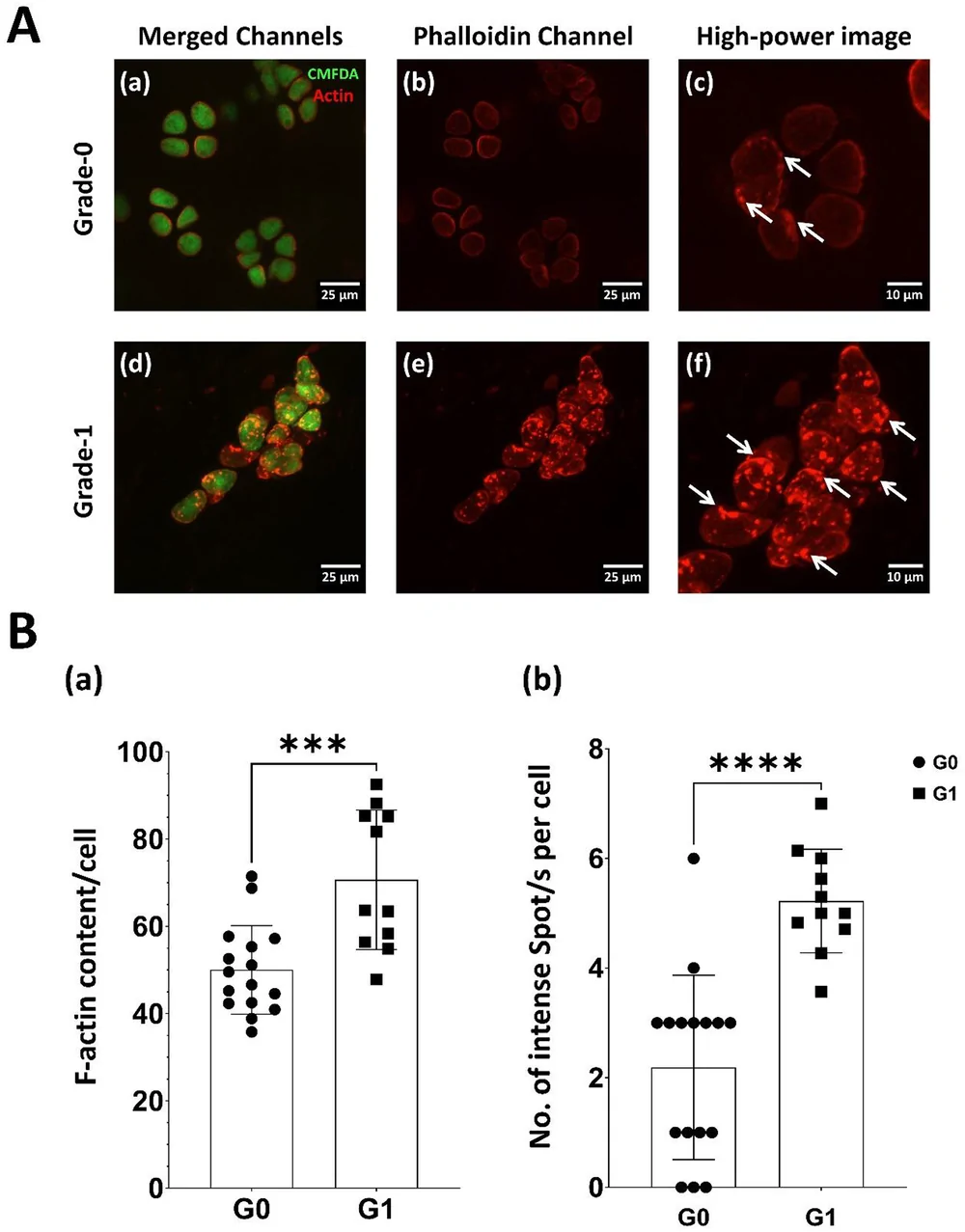
Comparison between F‐actin levels and its distribution between clustered chondrocytes in G0 and G1 femoral head cartilage. Panel (A) shows examples of clustered in situ chondrocytes and their F‐actin distribution in G0 and G1 cartilage with low (a, b) and (d, e) (bar = 10 μm) and high (c, f) (bar = 25 μm) power magnification images, respectively. Arrows identified examples of F‐actin localization and content. Panel (B) (a) shows the highly significant increases in average F‐actin fluorescence/cell and (b) the number of intense areas/cell between chondrocyte clusters in G0 and G1 cartilage (see Section 2 for image analysis methods). Each data point represented the averaged data from one image. Pooled data from (G0) – *N*(*n*) = 3(16) and (G1) – *N*(*n*) = 6(11); with at least three images analyzed for each femoral head.

### 3.3 Chondrocytes With Abnormal Morphology

A major feature of non‐degenerate and mildly degenerate human cartilage was the presence of morphologically‐abnormal in situ chondrocytes with fine cytoplasmic processes [14, 15]. In axial images, distinct heterogeneous labeling of F‐actin was observed in cytoplasmic process/es of morphologically‐abnormal in situ chondrocytes within both G0 and G1 cartilage (Figure 5A – (a, b, d, e)). At higher magnification, the areas of increased F‐actin content were clearly evident, particularly within cytoplasmic processes of both G0 and G1 cartilage (Figure 5A – (c,f), respectively). The average F‐actin level per cell within morphologically‐normal compared to cells with cytoplasmic processes of both grades was significantly greater (G0 – 59.6 ± 22.2 FI vs. 77.8 ± 21.4 FI; *p* < 0.0001, and G1 – 64.5 ± 22.4 FI vs. 69 ± 21.6 FI; *p* = 0.0158; Figure 5B – (a)). The F‐actin content within the cell body and its associated cytoplasmic process/es were assessed separately on a cell‐by‐cell basis. In both cartilage grades, the F‐actin of the cytoplasmic process/es was higher in G0 (35.4%; *p* < 0.0001) and G1 (13.1%; *p* = 0.0152) compared to their cell body, and also between cartilage grades (Figure 5B – (b)). These results indicated the increased accumulation of F‐actin within the cytoplasmic process compared to the cell body.

**Figure 5:**
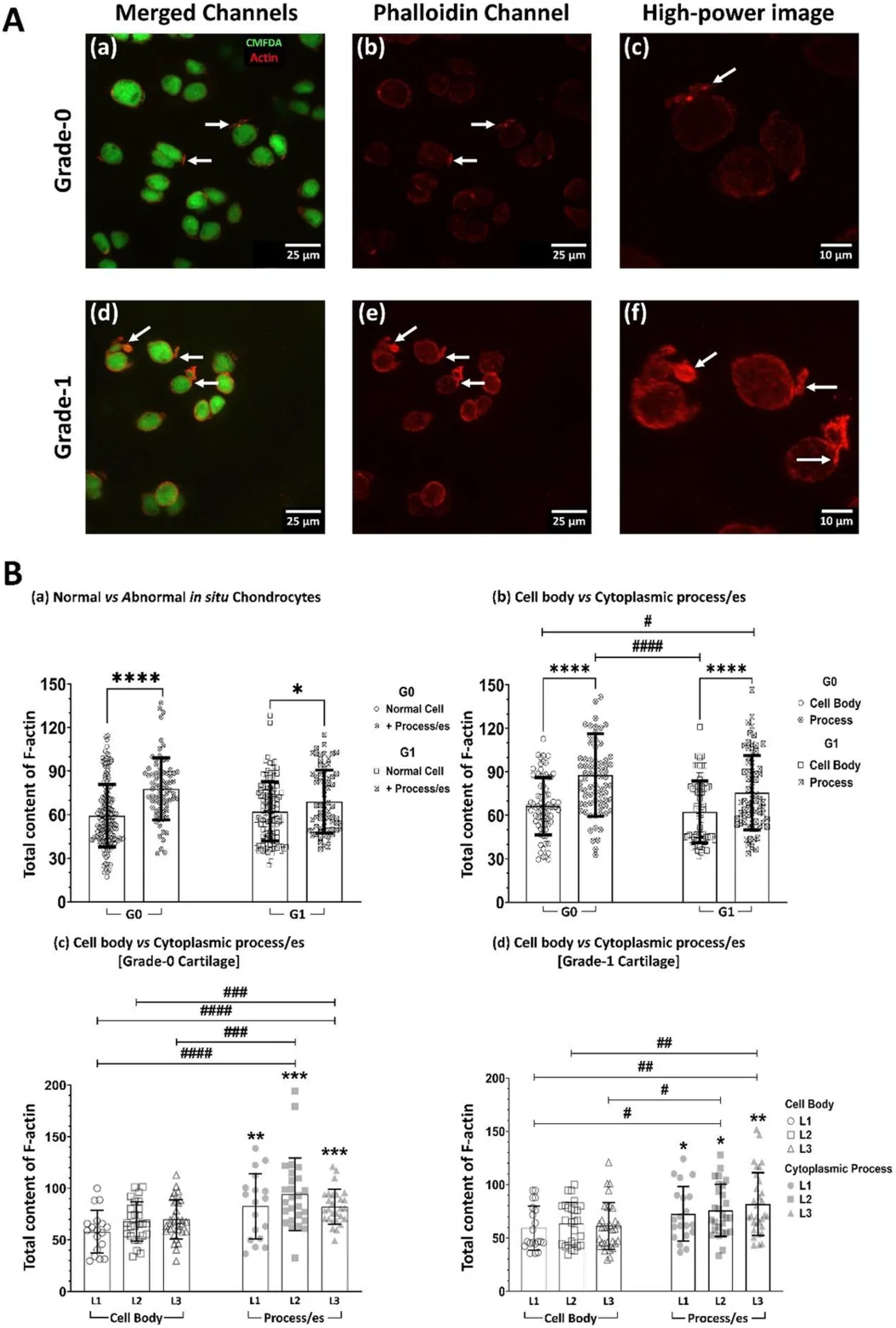
F‐actin within the cell body and cytoplasmic processes of chondrocytes in G0 and G1 femoral head cartilage. Low [(A) – G0 (a, b); G1 (d, e)] and high [(A) – G0 (c); G1 (f)] magnification images demonstrating the presence of F‐actin in the cortical region of the cell body, and extensive labeling within the cytoplasmic process/es identified using white arrows (A (c, f)). Panel (B) (a) shows fluorescently‐labeled F‐actin in comparison between morphologically‐normal versus morphologically‐abnormal in situ chondrocytes in both grades. Panel (B) (b) shows fluorescently‐ labeled F‐actin level compared between the cell body and cytoplasmic process/es within (paired *t*‐tests) and across (unpaired *t*‐tests) grades. Comparisons of fluorescently‐labeled F‐actin levels between the cell body and cytoplasmic process/es, classified according to process length (L1, L2 and L3; see Section 2), are shown for G0 and G1 cartilage (Panel B, c and d, respectively; *N*(*n*) = 8(75) and 9(70), respectively; ANOVA, p < 0.05).

The cytoplasmic process/es of morphologically‐abnormal in situ chondrocytes in G0 and G1 cartilage were sub‐classified into three groups depending on their length (L1, L2, L3; see Section 2). For both cartilage grades, F‐actin within the cytoplasmic process/es of the three groups (L1, L2, L3) was significantly greater (G0 – *p* = 0.0043; *p* = 0.0007; *p* = 0.001, respectively, and G1 – *p* = 0.037; *p* = 0.022; *p* = 0.0014, respectively; Figure 5B – (c,d), respectively) compared to their corresponding cell body. When the F‐actin of cytoplasmic process/es of differing lengths was compared to the cell body (i.e., process/es of L1 vs. the cell body of L2 and L3), for all three categories, F‐actin levels were higher (L2 – *p* < 0.0001; *p* = 0.0004, Figure 5B – (c) and L3 – *p* < 0.0001; *p* = 0.001, Figure 5B – (d)). For the L1 group, there was, however, no difference (*p* = 0.066; *p* = 0.066, Figure 5B – (c,d)) between the cartilage grades. Taking these results together, for both cartilage grades, F‐actin was significantly enriched within the chondrocytes with abnormal morphology compared to morphologically‐normal in situ chondrocytes, particularly within the cytoplasmic process/es compared to the cell body.

The previous results suggested the preferential localization of F‐actin in the cytoplasmic processes compared to the cell body, but did not identify its distribution within the processes themselves. Further investigations were performed, as it might have been expected that this would parallel the active development of these processes. Examples of F‐actin distribution are shown in Figure 6A – (a–d), illustrating its localized content associated with the developing cytoplasmic processes and its presence at the base and tip of more established processes. To determine the localization of F‐actin within the various parts of the cytoplasmic processes, they were divided into three sites (base, body, tip; see Section 2). The F‐actin content in each region was expressed as a percentage of the total F‐actin content within the whole cytoplasmic process.

**Figure 6:**
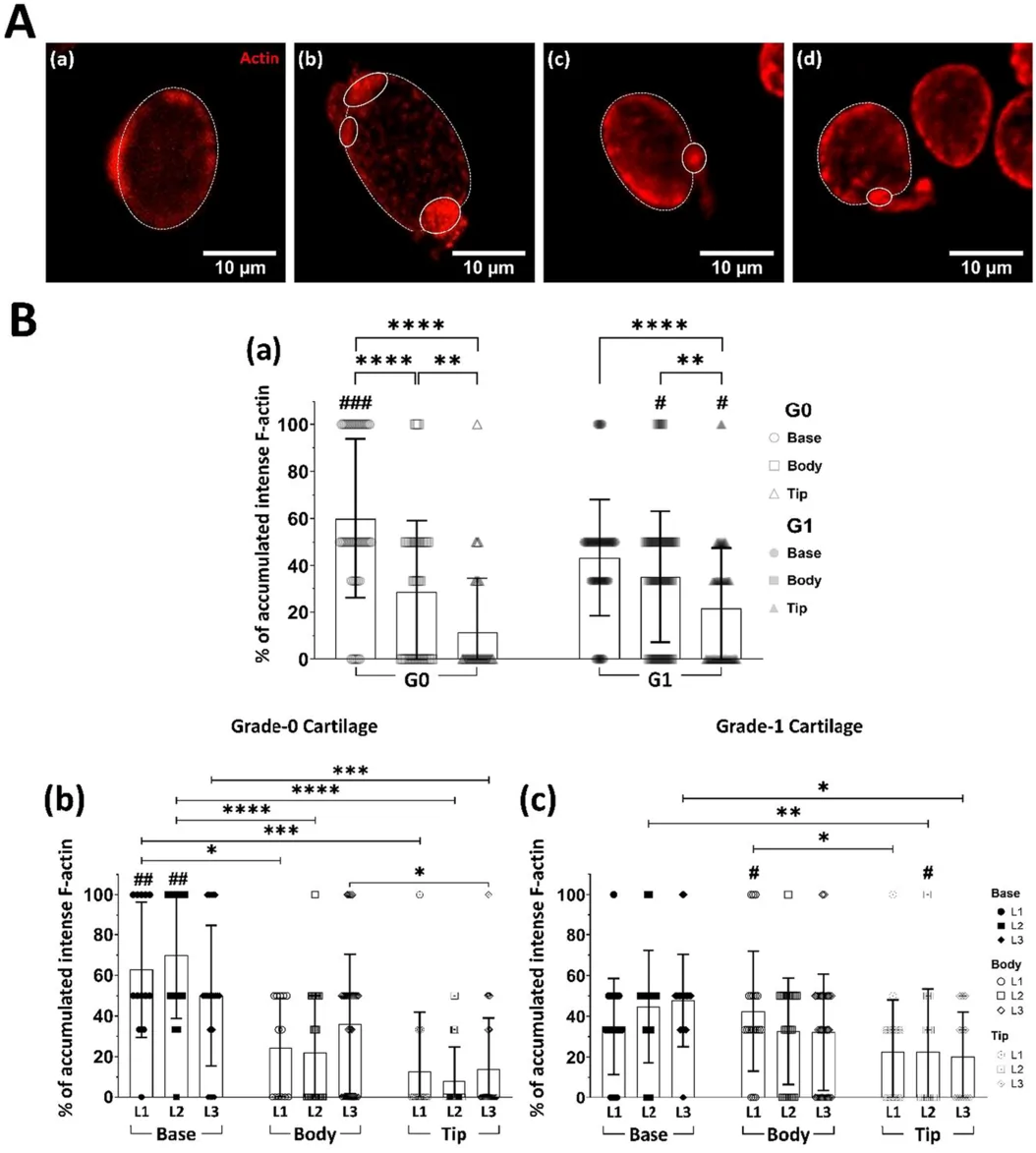
F‐actin localization within the cytoplasmic process/es of in situ chondrocytes in G0 and G1 cartilage. Panel (A) shows examples of F‐actin associated with (a) a bulge, (b) a small cytoplasmic protrusion, (c) and (d) different F‐actin distributions within the cytoplasmic process associated with changing chondrocyte morphology. Panel (B) shows pooled data of the comparisons between the F‐actin content in the base, body, and tip (expressed as a percentage of the total F‐actin content in the process) within all the cytoplasmic process/es of chondrocytes between, and across, G0 and G1 cartilage (panel B (a)). Panel B (b and c) shows the separation of cytoplasmic process/es into groups according to their length as described (L1, L2, L3, see Section 2) for cells in (b) G0 and (c) G1 cartilage. G0 data were from *N*(*n*) = 8(63); G1 data from *N*(*n*) = 9(74).

For abnormal chondrocytes in G0, the majority of F‐actin was at the base of the cytoplasmic process/es compared to the body and tip (60% ± 4.1%; *p* < 0.0001; Figure 6B – (a)). In contrast, the F‐actin content within the cytoplasmic process/es in G1 was significantly higher at the base and body (43.2% ± 2.9%; 35.1% ± 3%, respectively) compared to that at the tip of the process/es (*p* < 0.0001; *p* = 0.0055, respectively Figure 6B – (a)).

Equivalent sites on the process/es between cells in G0 and G1 cartilage were compared, and this indicated that the highest level of F‐actin in G0 was at the base (Figure 6B – (a); *p* = 0.0001). This differed from the F‐actin distribution for cells in G1 cartilage, where it was less at the base, but greater at the body and tip (*p* < 0.05; Figure 6B – (a)). This suggested that for chondrocytes with processes in mildly degenerate (G1) cartilage, the F‐actin content was greater at the body and the tip, in contrast to the cells in G0 cartilage, where the F‐actin was mainly in the base close to the area where a cytoplasmic process would be expected to develop.

Further investigations were conducted on F‐actin distribution of cytoplasmic process/es of various lengths. In G0 cartilage, the F‐actin of in situ chondrocytes with L1 and L2 cytoplasmic process/es was evident at the base (64.6% ± 10.7%; 68.3% ± 6%, respectively) of the cellular protrusion compared to the body and tip sites (*p* < 0.05; *p* < 0.0001, respectively, Figure 6B – (b)). However, L3 cytoplasmic process/es exhibited a different F‐actin localization, as the base 50% ± 6.4% and body 36.2% ± 6.4% shared the highest percentage compared to that of the tip (*p* = 0.0001; *p* = 0.0226, respectively, Figure 6B – (b)).

For the chondrocyte protrusions of L2 and L3 in G1 cartilage, F‐actin was significantly less in the base compared to the base of equivalent cells in G0 cartilage (*p* = 0.0038), whereas the body was not different (*p* > 0.05), however, it was increased in the tip of the processes (*p* = 0.044, respectively, Figure 6B – (c)). This suggests a reduction in F‐actin at the base of the process as the tip extended into the ECM. Finally, cytoplasmic processes classed as L1 in G1 cartilage did show a significant increase in F‐actin between the body and tip sites compared to matching cells in G0 cartilage (*p* = 0.0183; Figure 6B – (c)).

In summary, the high percentage of F‐actin labeling at the base of the cytoplasmic process/es in both cartilage grades suggested that the redistribution of F‐actin along the cortical ring was an early stage in the formation of cytoplasmic processes and was associated with changes to cell morphology. Furthermore, these results suggested that within the cytoplasmic process/es, the fluorescently‐labeled F‐actin repositioned to drive the protrusion of the cytoplasmic process/es, with the labeling decreasing at the base, and progressing to the body and tip of the processes.

### 3.4 The Response of In Situ Chondrocytes to Hypo‐Osmotic Pressure

The previous results suggested that the development of the cytoplasmic processes in chondrocytes of abnormal morphology was an “active” event involving the redistribution of F‐actin from a base in the cortical ring to the tip. However, there was the possibility that the cytoplasmic processes were a “passive” event as a result of changes to the ECM due to GAG/matrix loss with cartilage age. If so, then reducing osmotic pressure would cause not only the cell body to expand, but also the cytoplasmic processes to swell/lengthen through the matrix. To test this, experiments were conducted to determine the response of chondrocyte volume, together with cytoplasmic process width and length, following a hypotonic challenge applied to the fluorescently‐labeled in situ chondrocytes. Mildly‐degenerate (G1) cartilage explants were chosen as they exhibited suitable cytoplasmic processes. Explants were exposed initially to a control culture medium of normal osmolarity (340 mOsm), and then challenged with a hypo‐osmotic medium (170 mOsm; see Section 2). Figure 7A – (a–c) shows low magnification axial images of chondrocytes prior to (labeled green) and following (labeled red) osmotic challenge, with the merged channels (Figure 7A – (c)) showing the difference. At higher magnification (Figure 7B – (a–f)), the changes are more clearly seen, with the emergence of the red channel (post‐osmotic challenge) being apparent in the merged channel as the chondrocytes increased volume and changed shape. Three variables were analyzed (cell volume, width, and length of the cytoplasmic processes) in the same cells prior to and after osmotic challenge (Figure 7C – (a–c)). Chondrocyte volume of human G1 cartilage was 680.6 ± 28.4 μm^3^ and increased significantly to (811.9 ± 32.6 μm^3^; *p* = 0.0027) following hypo‐osmotic challenge (Figure 7B – (a–c) and Figure 7C – (a)). Similarly, the width of the cytoplasmic process/es (3.1 ± 0.4 μm) increased to 3.7 ± 0.5 μm (*p* < 0.0001; Figure 7B – (d–f) and Figure 7C – (b)). However, the length of the cytoplasmic process/es (31.7 ± 5.7 μm) did not change significantly in response to low osmotic pressure (31.8 ± 5.8 μm; *p* > 0.05; Figure 7B – (a–c) and Figure 7C – (c)). These results show that while there was swelling of the cell body and either side of the process, this did not occur in the region of the tip of the processes. This supported the notion that the development of the processes was an “active” event with F‐actin playing a key role.

**Figure 7:**
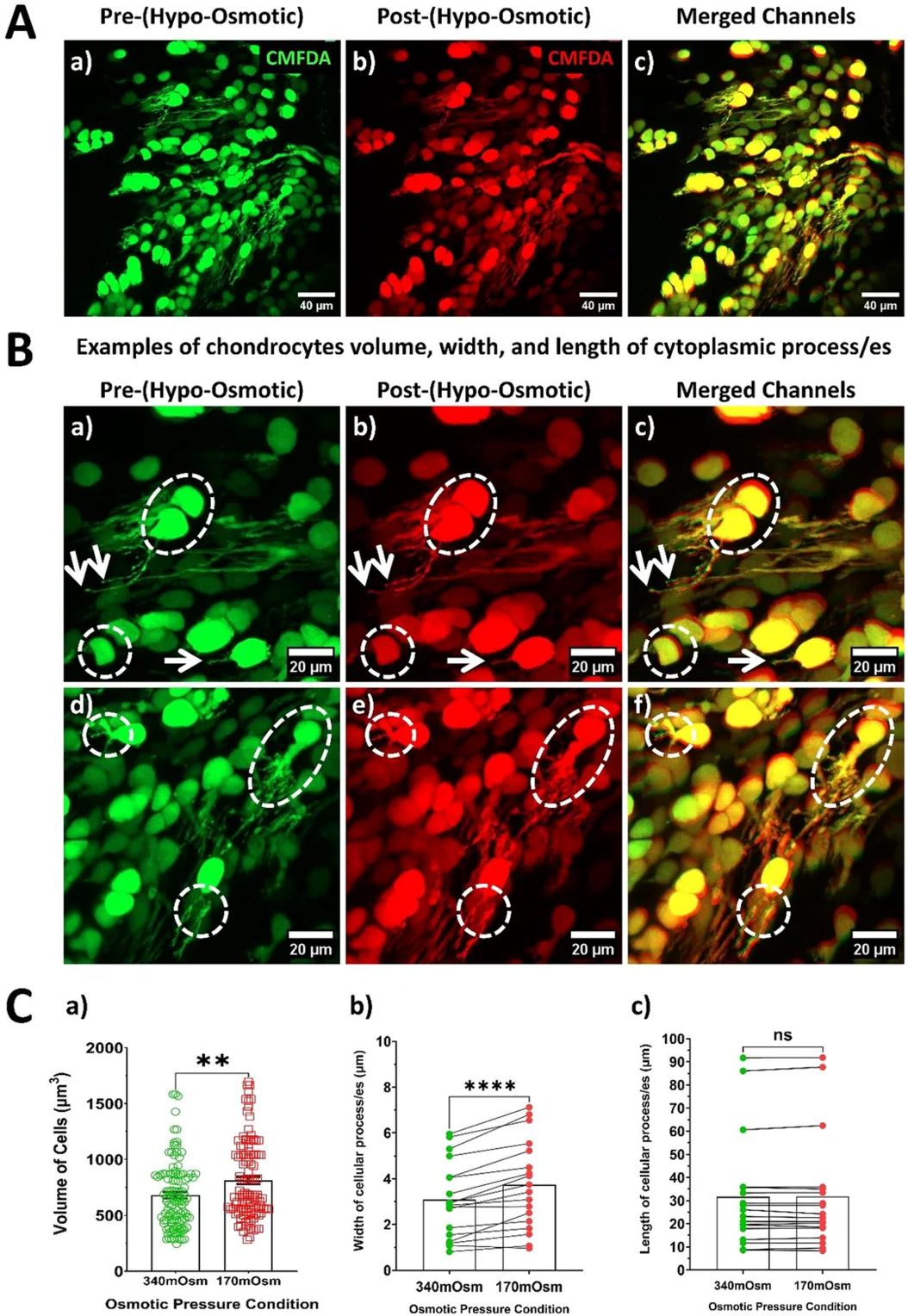
Changes to chondrocyte cytoplasmic processes in response to hypo‐osmotic pressure. (A) Low magnification images with examples of single abnormal in situ chondrocytes from mildly degenerate (G1) human femoral head before (a) and after (b) hypo‐osmotic pressure, and with both merged in one image (c). Scale bar = 40 μm. Cells were stained with CMFDA (green) for all channels (a–c), however, the color was changed (see Section 2 for details). Panel (B) (a–c) High magnification images were acquired to show examples of the changes to the individual cells (broken white circles/ellipses) with white arrows identifying individual cytoplasmic process/es. Panel (B) (d–f) represents examples of changes to the width of processes identified with broken white circles/ellipses. Scale bar = 20 μm. Panel (C) represents a comparison between the chondrocyte (a) volume, (b) width, and (c) length of cytoplasmic process/es before (normal osmotic pressure 340 mOsm) and after (low osmotic pressure 170 mOsm). Data from *N*(*n*) = 3(3) with a total of 18 cells analyzed.

## 4 Discussion

The simultaneous visualization of fluorescently‐labeled in situ human chondrocytes and associated F‐actin cytoskeleton using CLSM revealed the close relationship between chondrocyte volume and morphology and this key cytoskeletal element. When comparing the properties of chondrocytes within non‐degenerate and mildly degenerate cartilage, cell swelling, proliferation, and the development of cytoplasmic processes were evident, and associated with marked changes to F‐actin content and cellular distribution. Of particular interest was the finding that in some cells, focal F‐actin labeling was present, particularly in the base and body of the cytoplasmic processes, suggesting that this might play an active role in the development and extension of the processes. To further explore whether these fine cytoplasmic extensions resulted from an active cellular mechanism or passively, hypo‐osmotic challenge experiments were conducted. These demonstrated the absence of any increase in the length of the processes despite clear swelling of the cell body in response to lowered osmolarity. These results, taken with the focal accumulation of F‐actin associated with the cytoplasmic processes, supported the notion that the formation of the processes involved the active remodeling of the F‐actin cytoskeleton rather than being due to the processes swelling “passively” into regions of reduced matrix stiffness.

Increased cartilage hydration and chondrocyte swelling are well‐recognized early changes occurring with OA [15, 41], and this would be expected to lead to changes to the chondrocyte's cytoskeleton. Previous studies on the effects of osmotic changes have largely been confined to the application of a rapid (seconds to minutes) osmotic challenge. These experiments demonstrated a decrease in F‐actin content attributable to the disassembly of cortical F‐actin with cell swelling, probably related to the activation of the signal transduction pathways stimulating regulatory volume decrease (RVD) [23, 25, 42, 43], initiating cell shrinkage and thus volume recovery. However, osmotic changes experienced by in situ chondrocytes in vivo within mildly‐degenerate (swollen) cartilage are very different, as the cells would be exposed to a longer‐term chronic and progressive reduction in tissue osmolarity [44]. For chondrocytes of normal morphology, there were significant increases in F‐actin content in addition to the number of IP and IA, especially for large cells (Figure 3). It seems reasonable to suggest that an overall increase in F‐actin content with chronic swelling may be expected in order to maintain the structural integrity of the cell membrane and its associations with the ECM.

There are interesting and relevant parallels between the observations reported here on F‐actin content and distribution and the response of isolated or in situ chondrocytes to the pro‐inflammatory cytokine IL‐1β [27]. IL‐1β treatment of in situ chondrocytes promoted the re‐distribution of F‐actin to focal and intense areas localized at the cell membrane and inhibited RVD, leading to cell swelling [28]. While the co‐localization between cytoskeletal elements, including F‐actin and ion channels, for example, aquaporin, directly involved in volume regulation has been established [25, 45], we do not know if local tissue levels of IL‐1β or the cell‐associated presence of this cytokine [46] are responsible for these changes in cytoskeletal organization. In any event, there is a continuing debate about the contribution of this cytokine as a key inducer of cartilage degeneration in OA [47, 48].

Chondrocyte “clustering,” where the cells proliferate within their lacunal space, is another well‐characterized abnormal cellular feature of (OA) cartilage [14, 19]. Previous studies on in situ human chondrocytes confirmed that the increase in cluster size was due to cell proliferation rather than cell swelling/hypertrophy [14]. Our results identified a significant rise in the level and distribution of F‐actin within proliferating in situ chondrocytes (Figure 4). It is thought that the penetration of factors (e.g., fibroblast growth factor) into a weakened matrix simulates the expression of proliferation‐associated genes (e.g., Ki67, CCND1), particularly where chondrocytes are close to surface fissures [19]. F‐actin polymerizes in parallel with the gene expression of signaling molecules, inducing the proliferation process [13]. Additionally, integrin clustering, where the F‐actin cytoskeleton is implicated, can initiate proliferation [10]. In agreement with this was the focal F‐actin distribution identified as intense “spots” (Figure 2A), which could be a point of assembly for focal adhesion receptors such as integrins [11] responsible for initiating cellular proliferation. Other studies have reported that F‐actin de‐polymerization using cytochalasin D [13] or adseverin deletion in the whole tissue [9] reduces proliferation. In any event, these results illustrate that caution should be exercised if F‐actin levels are taken as a baseline “housekeeping” protein [49], as its levels will vary depending on the degree of cartilage degeneration and thus changes to chondrocyte morphology/proliferation.

For morphologically‐abnormal chondrocytes, there was strong punctate F‐actin labeling associated with cytoplasmic processes (Figure 5). In both G0 and G1 cartilage, levels were significantly greater for the average fluorescence/cell compared to normal cells (Figure 5B – (a)) and increased in cytoplasmic processes compared to the cell body (Figure 5B – (b)). A more detailed analysis of the F‐actin distribution within the cytoplasmic processes was performed by assessing F‐actin levels at the base, body, and tip of the processes (Figure 6). For these cells in G0 cartilage, levels at the base were greater than those at the cell body or the tip of the processes, however, in the morphologically‐abnormal chondrocytes in G1, F‐actin was elevated at the body and tip (Figure 6A). This suggests a progressive and active redistribution and/or production of F‐actin reflecting the development of the cytoplasmic processes. Analysis of punctate F‐actin labeling did not, however, suggest that there was a relationship with cell orientation, and the direction of the cytoplasmic processes appeared to be random (M. Al Garni unpublished observations). Future studies on the simultaneous visualization of F‐actin and G‐actin would provide a valuable insight into actin polymerization dynamics during cartilage degeneration.

The development of cytoplasmic processes appears to be closely related to other changes in chondrocytes and their surrounding matrix. For example, there is a significant correlation between the number of processes/cell and cell‐associated IL‐1β labeling, particularly for processes of < 5 μm in length [46]. In addition, there is a decrease in lacunar coverage and thickness of pericellular type VI collagen, especially for those chondrocytes with a large average length of processes [46]. Furthermore, cell‐associated levels of ADAMTs‐4 (A Disintegrin and Metalloproteinase with Thrombospondin Motifs‐4) measured on single in situ human chondrocytes by immuno‐fluorescence were increased in abnormal chondrocytes compared to morphologically normal or “clustered” cells [50]. Taking these results together, the development and penetration of cytoplasmic processes may result from localized pericellular GAG and lacunar type VI collagen degradation, providing a path through the matrix. An increase in levels of cell‐associated collagen type I has also been identified in human chondrocytes with cytoplasmic processes [51]. This is potentially important because this collagen is a recognized marker of chondrocyte de‐differentiation to a fibroblastic phenotype [10, 52]. Other studies have identified that degradation and abnormal metabolism of collagen are key pathological events in OA [53]. Thus, the formation of these delicate cytoplasmic processes, together with the loss of the chondrogenic phenotype, could initiate the focal development of a weak fibro‐cartilaginous ECM characteristic of early OA. Recent work by Pepper et al. [54] suggested a link between early morphological changes to chondrocytes and their susceptibility to subsequent inflammatory events. Increased chondrocyte spreading/morphological changes are observed in 3D cultures where matrix stiffness was reduced [55]. The authors emphasized the importance of maintaining chondrocyte morphology and the optimal extracellular microenvironment during the early stages of OA [54].

To determine whether the lengthening of the cytoplasmic process/es of abnormal in situ chondrocytes reflected a passive response due to a compromised local ECM, hypo‐osmotic challenge experiments were undertaken. The expectation was that the cell body and length of the processes should swell passively, however, the ends of the processes would not, as these were the result of the “active” development of the processes. Exposure of mildly degenerated (G1) cartilage to low osmolarity stimulated chondrocyte swelling and thickening of the cytoplasmic processes, but did not significantly affect their length (Figure 7C – (a,b), respectively). This suggested that the matrix surrounding the cell and body of the process lacked sufficient mechanical integrity to resist volume‐driven deformation; however, the lengthening of the processes was still restricted by the stiffer normal matrix. It is also possible that matrix pore size, rather than stiffness, could be the true limiting physical factor for the penetration of the processes [37].

For the processes to develop, it seems more plausible that there must be local enzymatic degradation of the matrix rather than “passive” extension. This interpretation is supported by the active recruitment and localization of the chondrocyte cytoskeleton involving F‐actin polymerization to permit the extension of the processes [12]. An interesting recent study suggested that human OA chondrocytes in 3D degradable hydrogels have a particular propensity to exert higher contractile forces and enhanced protrusive activity [56], compared to non‐OA chondrocytes. The present use of CLSM imaging of fluorescently‐labeled single in situ chondrocytes and their F‐actin content and its distribution revealed microscopic changes to chondrocytes within non‐degenerate and mildly degenerate human cartilage. While some of these changes might be considered small – albeit statistically significant, and have little bearing on the overall properties of the ECM, it must be remembered that OA progression is slow and occurs over years/decades. Thus, the cumulative effect of the aberrant matrix metabolism by abnormal chondrocytes could be substantial, and understanding the changes described here could offer targets for slowing the process. In summary, the results presented here highlight the unique properties of these abnormal human chondrocytes, which may possess harmful characteristics that are damaging to the otherwise healthy, mechanically resilient matrix.

## Data Availability

The data that support the findings of this study are available from the corresponding author upon reasonable request.
